# Fast Distributed Dynamics of Semantic Networks via Social Media

**DOI:** 10.1155/2015/712835

**Published:** 2015-05-17

**Authors:** Facundo Carrillo, Guillermo A. Cecchi, Mariano Sigman, Diego Fernández Slezak

**Affiliations:** ^1^Laboratorio de Inteligencia Artificial Aplicada, Departamento de Computación, Ciudad Universitaria, 1428 Buenos Aires, Argentina; ^2^Computational Biology Center, T. J. Watson Research Center, IBM, P.O. Box 218, Yorktown Heights, NY 10598, USA; ^3^Universidad Torcuato Di Tella, Avenida Figueroa Alcorta 7350, 1428 Buenos Aires, Argentina

## Abstract

We investigate the *dynamics* of semantic organization using social media, a collective expression of human thought. We propose a novel, time-dependent semantic similarity measure (TSS), based on the social network Twitter. We show that TSS is consistent with static measures of similarity but provides high temporal resolution for the identification of real-world events and induced changes in the distributed structure of semantic relationships across the entire lexicon. Using TSS, we measured the evolution of a concept and its movement along the semantic neighborhood, driven by specific news/events. Finally, we showed that particular events may trigger a temporary reorganization of elements in the semantic network.

## 1. Introduction

Language has been studied from a wide variety of perspectives, including aesthetical, evolutionary, and mathematical [1]. An essential function of language is to convey meaning, endowing speakers, writers, listeners, and readers with the ability to encode and decode behaviorally relevant information in auditory and visual tokens. Any attempt to quantify the intuitive notion of meaning involves, in particular, a characterization of the semantic similarity between words. Semantic quantification has in fact become a pressing issue in machine learning and linguistics, with growing applications ranging from automated recommendation and customer service systems [2] to speech analysis in psychiatry [3].

An extensive body of literature has lent support to the hypothesis that frequency of cooccurrence of words and semantic similarity between the corresponding concepts are codetermined, including psychophysics of word association [4] and coextensivity of neural activation of associated words [5, 6]. Typically, subjects associate faster two related words than unrelated ones, that is, if they belong to the same category [7]. This feature can be quantified by analysing text databases looking for cooccurrence of words and defining semantic spaces that may cluster words coming from the same category [8]. This kind of approaches grows beyond computational capacity very easily, and so several dimensionality reduction techniques have been applied to treat these problems (e.g., Latent Semantic Analysis [9] or topic models [10]).

A widely used computational correlate of this hypothesis is Latent Semantic Analysis and related measures, which estimate semantic similarity on a vector space defined by word frequencies [9]. Similarity, measured in the newly defined vector space, shows accordance with several experiments such as word synonyms [11], conceptual match between topic knowledge and text information [12], or measuring the coherence of texts [13].

Frequencies have been evaluated from large text corpora [11] and, recently, from web-based document aggregation [14]. These methods have been very successful in providing a stationary map of word similarity. However, the organization of the lexicon changes dynamically both in the time scale of human development [10, 15] and in the longer time scale of cultural transformation [16, 17]. Our goal is to investigate the* dynamics* of semantic organization, developing a consistent measure of word similarity computable in narrow temporal windows.

In order to estimate semantic spaces in short periods of times, Latent Semantic Analysis [9], topic models [15], or Word2Vec [18] must recalculate the whole semantic space for each time window. At each instant, a* representative* corpus of text must be collected to train the corresponding semantic space. Instead, we focus on estimating the semantic space by studying the activity in the social network with a similarity measure computationally cheap to calculate.

To this aim we implemented a word cooccurrence measure based on the social networking and microblogging service Twitter. There is growing interest in the analysis of Twitter timelines (e.g., [19, 20]), driven by the ability of this corpus to identify with exquisite temporal resolution the collective expression of human thought. The fundamental difference between previous studies and our work is that our analysis is not based on tracking the “trending topics” defined by a handful of words [21]. Instead we assume that semantic content is inherently distributed but with a structure that is subject to a “topic-driven” evolution and, possibly, its own internal dynamics.

## 2. Methods

### 2.1. Twitter Semantic Similarity Algorithm

Normalized Google distance proposes a method for measuring similarity between words analysing cooccurrence relative to their individual frequencies in the World Wide Web [22]. Current implementations fail because of accuracy of occurrence estimation of queries. Moreover, Google corpus provides an almost-static database with no fine-grain temporal information. Based on these previous results, we developed Twitter Semantic Similarity (TSS), estimating similarity between words with a high-resolution temporal precision.

Twitter is an online platform for microblogging with messages 140-character long, with more than 50 million tweets per day. Twitter provides access to automatic crawling of tweets, at a limited rate, approximately 180 queries every 15 minutes, through the Twitter API (here we used Twitter API version 1.1). To search through Tweets database a search key must be provided and optional parameters are available. The search key is a string with up to 1,000 characters including spaces. Each query may return a maximum of 100 tweets, with their time stamps. Due to this limitation, it is not possible to retrieve every tweet containing a word, or bag of words, or all tweets framed in a period of time. Instead, we propose the use of the provided time stamp of tweets to calculate the velocity of production of tweets containing the word. Thus, we estimate the frequency of a word in Twitter from its velocity of occurrence.

Considering the word *w* and the time stamp series {*τ*_*i*_(*w*)} of *N* tweets containing *w* (any number of occurrences of *w* in the tweet), the frequency Φ(*w*) may be estimated as the average time between tweets in this series. Formally,

(1) $$\begin{array}{c} \Phi \left(w\right)={\left(\frac{\sum_{i=1}^{N-1}\left(\tau_{i+1}\left(w\right)-\tau_{i}\left(w\right)\right)}{N-1}\right)}^{-1}. \end{array}$$

The size of the time stamp series, *N*, is an adjustable parameter that depends on the Twitter API, with a limit in 100 tweets per query. We use *N* = 30. Thus, for a word *w*, velocity is estimated as the mean of the difference between consecutive time stamps of the last 30 tweets containing *w*.

We similarly estimate the frequency of cooccurrence of two words *w*_1_ and *w*_2_, Φ(*w*_1_∧*w*_2_), from the production of tweets that contain both, regardless of their relative order within the tweet. We define the TSS between two terms *w*_1_ and *w*_2_ as

(2) $$\begin{array}{c} \text{TSS}\left(w_{1},w_{2}\right)={\left(\frac{\Phi \left(w_{1}\wedge w_{2}\right)}{\max\left(\Phi \left(w_{1}\right),\Phi \left(w_{2}\right)\right)}\right)}^{\alpha } \end{array}$$

with *α* being a scaling factor (we use *α* = 1/4 obtaining a good scale). In the case that *w*_1_ and *w*_2_ are not present in Twitter, we define TSS = 0.

No preprocessing phase is needed; for example, search terms may be one word, multiple words, or hashtags. Note that terms may be surrounded by quotations marks, searching the multiple-word term in the Twitter API.

### 2.2. Common Semantic Categories

To evaluate the TSS algorithm, we defined three semantic categories containing a list of words. These categories were chosen as standard intuitive categories from common objects or concepts of everyday life:

 
*Fruits*: apple, banana, blackberry, blueberry, cherry, coconut, grape, kiwi, lemon lime, mango, melon, watermelon, orange, tangerine, papaya, passion fruit, peach, pear, pineapple, pomelo, raspberry, and strawberry.
 
*Animals*: bull, cow, chicken, donkey, goat, horse, pig, rabbit, sheep, dolphin, shark, octopus, turtle, bird, eagle, mouse, owl, bear, bat, dog, cat, fly, ant, and tiger.
 
*Colors*: blue, green, red, yellow, orange, black, white, pink, brown, fuchsia, grey, purple, violet, and golden.

### 2.3. Group Organization Performance Based on TSS

To study global reorganization of semantic network, we implemented a performance value to quantify how TSS captures the World Cup group organization. We calculated the TSS value between all combinations of the 32 qualified countries, that is, the 32 × 32 symmetric similarity matrix of country pairs. For each country, we selected the three countries more TSS-similar to it and assigned a performance value at every instant. If the three countries belonged to the same World Cup group we assigned a value of 1.0; if two of them belonged to the same group, we assigned a value of 2/3; if only one of them belonged to the same group, we assigned a value of 1/3; and if none belonged to the group 0 was assigned. We report the TSS performance as the mean value of the performance for every country at each instant (Figure 3(b)).

### 2.4. Classifiers and Cross-Validation

In the machine learning literature, a classifier is an algorithm that assigns labels to new incoming data based on a training dataset. A classifier has two implementation stages: (1) the training phase, consisting in learning the underlying patterns of the data and its labels, with many techniques available for this pattern recognition; (2) a test phase, where new data, not used for training, is labeled based on trained classifier. For studying the common semantic categories we used *K* ^∗^ [23] and for the reorganization of the countries network due to the World Cup draw, we used the Naive Bayes classifier [24].

To assess performance of classifier, cross-validation provides a validation technique. *k*-fold cross-validation consists of partitioning the training data into *k* subsets of the same size, using *k* − 1 samples as training data and testing the remaining item. This process is repeated for the *k* subsets, that is, the *k*-fold. This process is repeated for the *k* subsets, and classifier performance is estimated as the average classification performance for all subsets.

## 3. Results

### 3.1. Stationary Semantic Organization via TSS

Our algorithm can compute a matrix of word similarity with a time resolution which varies between days and can go for certain subset of the matrix of cooccurrences within the range of minutes. Before inquiring how concepts evolve in time for a specific set of experiments we verify that Twitter Semantic Similarity (TSS) as a stationary measure satisfies a series of validations that a well-behaved measure of semantic similarity is expected to pass.

First, we validated that TSS yielded similar results to well-documented measures of word similarity: cosine distance based on Latent Semantic Analysis [9] and standard measures based on Wordnet [25].

To this aim we selected 102,000 pairs of words (chosen randomly from the 1500 more frequent nouns) and computed their similarity based on Wordnet, LSA, and TSS. TSS showed a very strong correlation of word similarity to LSA (*ρ* = 0.2199, *p* = 0) and to several measures based on Wordnet: (1) shortest path that connects senses using hypernym and hyponym relation (*ρ* = 0.298, *p* < 10^−275^); (2) information-theoretic definition by Lin [26] (*ρ* = 0.15, *p* < 10^−67^); and (3) similarity based on information content by Resnik [27] (*ρ* = 0.157, *p* < 10^−74^).

Different algorithms of word similarity have been evaluated using TOEFL synonym tests [11, 28]. We analyze if TSS may discriminate synonyms from random pair of words. We selected 86 words from a TOEFL practice web page (TOEFL vocabulary words: http://toeflvocabulary.com/) and paired them with one synonym and 25 no-synonym words obtained from the 100K pairs of most frequent nouns. For each of the 86 words (*w*), we calculated the TSS and LSA value for all 26 options, obtaining 2 vectors *d*_TSS_^*w*^, *d*_LSA_^*w*^ of 26 components each. If we sort *d* ^*w*^ in increasing order, semantic measure should show the *w*-synonym words last in the list; that is, most similar words, higher similarity values, should be the synonyms. Mean position of synonyms in *d*_LSA_^*w*^ showed 〈*d*_LSA_^*w*^〉 = 0.602 ± 0.328, in concordance with previous results that state 60% of performance in TOEFL vocabulary exam [28]. With this result, synonyms are close to the middle in the ordered list, and so TOEFL exam should fail. On the other hand, for TSS we obtained that *d*_TSS_^*w*^ showed a higher value, 〈*d*_TSS_^*w*^〉 = 0.871 ± 0.204, which is significantly higher than the null hypothesis (the uniform distribution over the interval) and the results obtained with LSA.

**Figure 1:**
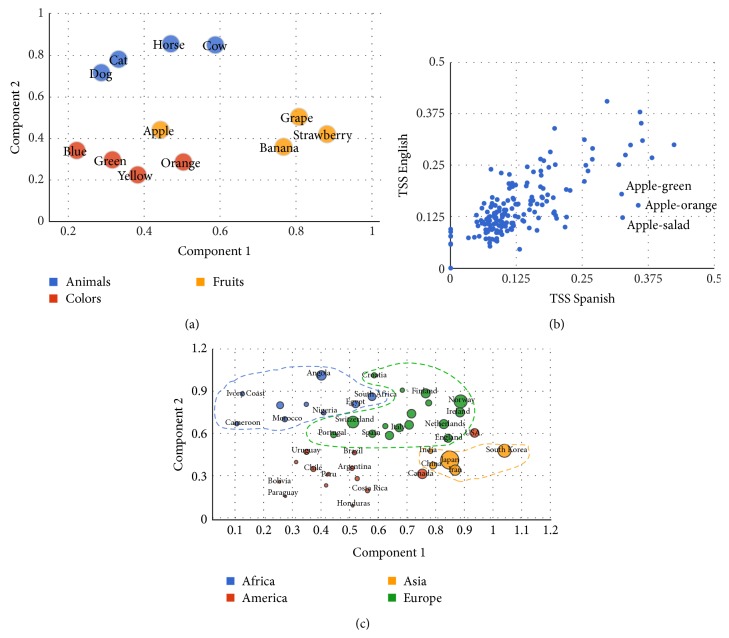
Stationary semantic organization using TSS. Panel (a) shows the clustering of 3 groups of words in semantic categories. Concepts belonging to the same category group together, with the exception of* apple*, closer to the colors cluster instead of the fruits cluster. Panel (b) shows the multilingual property of TSS, comparing 190 pairs of concepts in English and Spanish with a strong linear correlation *ρ* = 0.74, *p* < 10^−29^. Outliers (points which depart from the diagonal) are signaled with arrows, corresponding to the polysemic behavior of word apple in English. Panel (c) shows how TSS captures geographical and geoeconomical measures of world organization (diameter of points represents each country's GPD per capita).

Second, we verified that typical and easily recognizable semantic clusters are well described by TSS. To this aim we generated 1000 sets of 12 words belonging to three semantic categories: fruits, animals, and colors (see methods for the complete list of words in each category). For each set, we calculated the TSS similarity submatrix and run a 10-fold cross-validation *K* ^∗^ classifier [23]. Performance for TSS was very well above chance (*K* ^∗^ classification of chance generated groups is approximately 0.3 ± 0.0001) with values of 0.6846 ± 1.6458 × 10^−4^ (on the same dataset, 0.5543 ± 1.8265 × 10^−4^ for LSA and 0.6736 ± 1.5419 × 10^−4^ for Wordnet). To exemplify the capacity of TSS to cluster words in semantic categories, we used multidimensional scaling (MDS) [29] to project the semantic network to the 2-dimensional plane (Figure 1(a)). This representative example shows that words belonging to the same category cluster together. This particular example shows finesse of the metrics above and beyond classifying in broad categories. For instance, animals are subdivided in two natural categories (cat-dog and horse-cow). Second, the word orange which refers to a fruit and to a color is mapped between the two corresponding clusters. Third, within the fruits, the word apple is misrepresented. We reasoned that this may be due to polysemy of apple which relates to the fruit and to the brand. To examine this hypothesis, we first measured the proximity of the word apple to other fruits in English and in Spanish. Results showed that manzana (the word for apple in Spanish) is closer to the fruit cluster than apple.

This observation also leads to a test for TSS. It is expected that TSS should show results which are broadly independent of language and which may show some discrepancies in words which may show polysemy specifically in one language. To examine this hypothesis we measured the TSS correlation (in 20 words of 4 categories: animals, colors, fruits, and foods) between Spanish and English. The correlation was highly significant (*ρ* = 0.7304, *p* < 10^−29^) and within this set the outliers (points which depart from the diagonal) were pairs of words relating apple to other concepts: apple-orange, apple-green, and apple-salad, which are more similar in Spanish than in English. This effect is probably caused by the existence of the company apple that produces a polysemic behavior in English (and not in Spanish) for the word apple. We performed the same comparison to examine the correlation between English and the following languages: Portuguese, German, French, Italian, and Japanese. In all cases the correlation was significant (*ρ* > 0.5884, *p* < 10^−27^).

The last test of validity for stationary measures of TSS is whether it can capture geographical and geoeconomical measures of world organization. To this aim we selected the English words of countries of Asia, America, Africa, and Europe and measure their TSS.

A two-dimensional projection of this similarity matrix (Figure 1(c)) shows that the measure can identify to a large extent continental and geographical organization of the countries. It can clearly parse South America, Europe, and Asia, while the separation of North American countries (US and Canada) is less precise.

To quantify this observation we ran a classifier to see whether TSS can be used to infer the continent to which a country belongs. This was done in independent sets of seventeen: five countries of Asia, Europe and South America, and Canada and the US. We used 10-fold cross-validation and *K* ^∗^ classifier and we obtained a performance above 90%.

The projection of the TSS of words referring to countries also made evident an organization based on countries wealth. The wealthier countries were organized in the center of the graph while more peripheral countries tended to be poorer (Figure 1(c)). To quantify this observation we performed a linear regression of Gross Domestic Product (GDP) as a function of distance to the mass center of the 5 wealthiest countries. Results showed a highly significant negative correlation (*ρ* = −0.58, *p* < 10^−4^).

In summary TSS is a well-behaved measure of stationary semantic similarity: (1) it covariates with well-documented measures of semantic similarity; (2) it identifies natural semantic categories and outliers within these categories; (3) it is consistent across languages; and (4) it can identify within the same data more than one classification parameter (wealth and location in geographical data).

### 3.2. Dynamics of Semantic Organization

According to most modern views, mental concepts arise as an emergent property of their interrelationships. Thus, a concept is defined by whom it relates to within the network [8, 9, 30–32]. Wordnet, LSA, NGD, and Word Association metrics have identified consistent regularities in semantic networks which ought to be emergent constructs of mental activity of societies through time. However, it is expected that the organization of concepts varies through time; to the extent that language reflects thought, these changes should be reflected, specifically, in the use of words [14, 16]. Here, we capitalize on the capacity of TSS to identify rapid temporal fluctuations in word similarity to examine and validate this hypothesis.

#### 3.2.1. Evolution of a Concept: What Do We Think When We Think of Light?

On December of 2013, temperatures in Buenos Aires rose to unusually high values and elevated the home demand of energy which led to a major collapse of the service. This crisis was densely spread between December 16 and 30 through a city with a population of more than 13 million people and resulted in major riots, protests, picketing, and manifestations. During these days, the feeling was that people in the city of Buenos Aires could hardly talk about matters unrelated to heat, power supply, and political crisis and the relation between these concepts. We reasoned at the time (while waiting for the power supply to come back) that this was a unique occasion to investigate in quantitative grounds the drift of a concept. Specifically we hypothesize that the concept “luz” (which in Spanish refers to light but also generically to electricity and power supply) would transiently drift from its* static* neighborhood of associated concepts (spark, lamp, lightning, soul, sun, idea, etc.) to a set of words which would reveal the political and social tension and conflict and struggle evoked. In other words, our hypothesis is that, during these days, when Buenos Aires people thought about light they were not thinking in illumination, creativity, clarity, and so on but rather in conflict, tension, riots, and so on.

To examine this hypothesis we first defined two sets of words. One contains the concepts commonly associated with light: luminosity, candle, spark, bulb, and creativity. These concepts were derived from wordassociation.org (http://wordassociation.org/search/). A second set was generated by asking fifteen participants about the set of words which they thought reflected a chaotic social scenario, from which we derived the highest 15 words.

**Figure 2:**
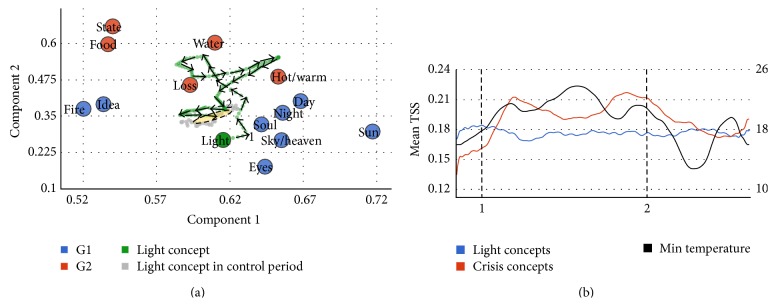
Evolution of the concept of light. Panel (a) shows the trajectory of the concept* light* around concepts commonly associated with light (blue points) and concepts reflecting chaotic social scenario (red points). Crisis period starts at point (1) and finishes in point (2). Grey points show the trajectory of light during the control period (3 months after crisis). Standard deviation around the two principal components of points in control trajectory is shown by the ellipse, showing that during crisis the light concept moves towards chaotic-associated words. Panel (b) shows the mean TSS of the concept of light for both groups (G1: light concepts, blue line; G2: crisis concepts, red line). Mean TSS between light and all words in G2 (chaos) showed a positive correlation with min temperature (black line).

We then had two sets of words (defined prior to any TSS measurement) and we could examine the hypothesis that the concept light should drift from a location close to Group 1 (Common Light Conceptual Neighborhood) to Group 2 during the crisis. We measured the location of the concept “light” within the semantic network 3 days before the crisis and up to 28 days after the start of crisis (Figure 2(a)). The trajectory shows a clear loop during the time of the crisis which departs from Group 1 words towards Group 2 words. As a control, we ran a second set of data more than one month after the crisis had been completed to measure typical fluctuations of the concept in similar time windows. Results showed that during this time the concept showed moderate fluctuations which on average were closer to G1 (and farther from G2) than during the crisis (G1: 0.23, G2: 0.22, *t*-test *p* < 10^−3^, df = 142).

To quantify this result we measured the mean TSS of the concept of light for both groups and the relation between the position of the concept in the graph and temperature (Figure 2(b)). Global data of power collapse was not available and since temperature correlates tightly with power demand this was the best estimate we had of collapse in power supply. As hypothesized, the mean TSS between lights and all words in the G2 (chaos) showed a positive correlation with min temperature (*ρ* = 0.5180, *p* < 10^−28^). Conversely, the mean TSS between light and all words in the G1 (typical light word associations) showed a negative correlation with min temperature (*ρ* = −0.1943, *p* < 10^−5^).

#### 3.2.2. Global Reorganization of the Semantic Network

In the previous section we showed that a single concept may drift in the semantic network in response to major social events. Here we investigate the possibility that a cultural event may reorganize the entire network in a different set of clusters. To this aim we capitalize on the example of country words described previously. This is a relatively simple domain of semantics, organized by reasonable principles of geography, culture, and economy. People may think of Sweden and Norway or Argentina and Uruguay as similar countries, revealing hence a sense of proximity in a semantic map. This intuition is testified by the results described in Figure 1(c) and the analysis described above. However, temporary political or contemporary events may reorganize this representation. A war, an international conflict, or even love (as in the marriage of the prince of Holland to an Argentinean wife) may temporary relate semantically to countries which before were thought and conceived as distant.

On December 6, the draw for the Football World Cup 2014 was held in Brazil. The World Cup is organized in eight groups of four national teams. The teams of each group play against each other to determine who will qualify to the final rounds of the best sixteen. From December 3 to December 14, we recorded the high temporal resolution TSS between 64 countries: 32 qualified national teams (QT) and 32 countries which did not participate trying to balance between continents, size, and population (Non-QT). The TSS three days before the draw already shows that QT are slightly more similar on average (〈TSS_3Dic_(QT)〉 = 0.11 ± 0.0089, 〈TSS_3Dic_(Non-QT)〉 = 0.07 ± 0.0085, *t*-test, *p* < 10^−5^, df = 2046).

It must be noted that countries attending to the World Cup do have socioeconomical, demographical, and geographical similarities. However, the dynamic nature of the formation of this cluster becomes clear when one analyzes the TSS matrix one hour before the draw. The similarity between the concepts representing the countries participating in the World Cup increases revealing that a clear cluster matrix shows a first stage (〈TSS_3Dic_(QT)〉 = 0.11 ± 0.0089, 〈TSS_6Dic_(QT)〉 = 0.33 ± 0.0087, paired *t*-test, *p* < 10^−200^, df = 1023).

An hour after the draw had been completed (it is important to bear in mind that a large fraction of the world population follows this draw in real time and with great expectation) the TSS matrix shows an internal structure within the concepts representing the 32 countries which reveals the outcome of the groups.

**Figure 3:**
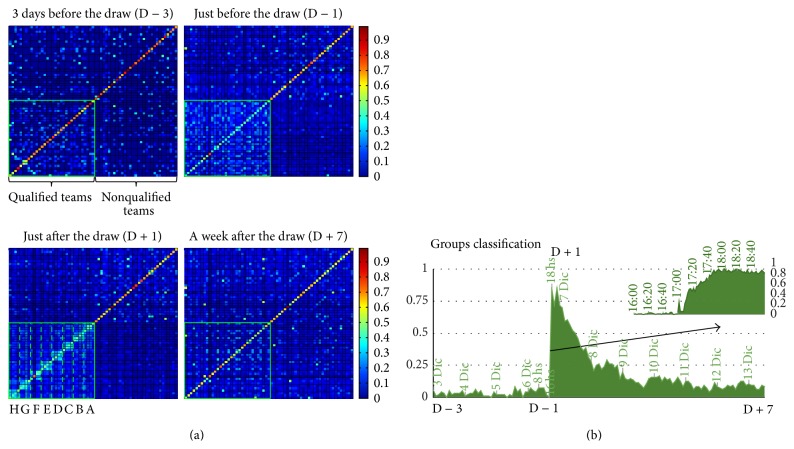
Reorganization of the semantic network caused by World Cup draw. Panel (a) shows the TSS between two sets of countries (32 qualified teams and 32 nonqualified teams) in four instants: 3 days before the draw (D − 3), just before the draw (D − 1), just after the draw (D + 1), and a week after the draw (D + 7). By normalizing matrix rows, panel shows internal structure within the concepts representing the 32 countries revealing the outcome of the groups draw. Panel (b) shows the classifier performance predicting the groups conformation every two hours. Between D − 3 and D − 1 classifier shows poor performance, with a rapid rise between D − 1 and D + 1 (see overlay for a two-minute resolution performance result), and the final decay after D + 1 until D + 7.

To quantify the information we run a classifier which, for each country, sought to identify the three other countries of its group based exclusively on TSS (see Methods for classification method). We generated 10000 random distance matrixes of all countries, showing a classification level, the chance level, of 0.1471 ± 0.0004. Before the draw (Figure 3(a)), classification showed a very low value (0.0329 ± 0.0034). This classification is below chance level due to the geographical dependence of TSS, as showed in Figure 1. It then ramps extremely rapidly to values close to 0.8 and fades down exponentially to values significantly above level previous to draw (0.1045 ± 0.0045, *t*-test, *p* < 10^−20^, df = 102) where it remains stable. We emphasize that this is a naive classifier that assumes that the three countries grouped with any given country are those with more similar TSS, hence ignoring all other reasons (geographical, economical, and social) why two countries might be similar.

**Figure 4:**
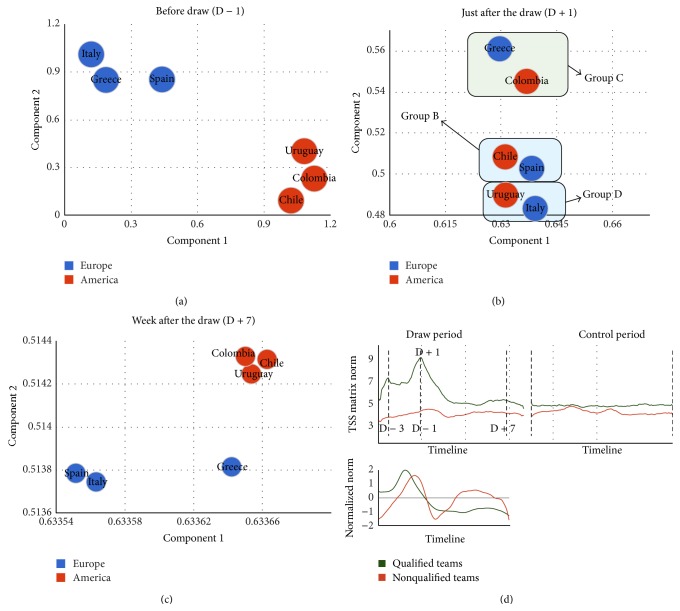
Episodic organization of the semantic network. We show an example of six countries in two continents and how World Cup draw reorganizes the semantic network from a continental organization (Panel (a)) to groups organization (Panel (b)) and back to the geographical organization (Panel (c)). Panel (d) shows how highlighted events impact all the semantic network, by calculating the matrix norm for TSS matrix at every instance, with each TSS matrix corresponding to TSS between words belonging to the same category (qualified and nonqualified). From D + 1 until the instant where the two series stop decreasing, these series show a stronger correlation than in other instants, implying that the World Cup draw modified the expected network (qualified teams) but also the nonqualified teams.

These results thus indicate that the temporary organization of the network is completely dominated by this episode completely overriding other constituting elements. To emphasize this idea, we followed the two-dimensional projection of six countries with the following properties: (1) Three are South American and three European; (2) after the draw, each of the South American countries is paired in a World Cup group with one of the European countries; we reasoned that (a) before the draw geographical similarity should dominate the network that should then be organized in two continental clusters and (b) just after the draw the network should organize in three clusters dominated by World Cup groups; and (3) after a few days the network should revert to its continental organization. The data showed a perfect continental classification (classifier: Naive Bayes, 2-fold cross-validation, 100% performance; see Methods for details) before the draw (Figure 4(a)) and 0% of group classification performance. Just after the draw, classifier showed a good performance of 83% of correct group classification (Figure 4(b), Naive Bayes, 2-fold cross-validation) and only 16% of geographical classification performance. A week after the draw, the geographical classification shows 83% of performance (Figure 4(c)) while group classification showed 0% performance.

Based on these results, we showed that the draw rearranges the qualified teams shifting from the geographic to World Cup's groups organization. However, we hypothesize that this highlighted event impacts all the semantic network, including the nonqualified teams subnetwork. To characterize this phenomenon we calculated the matrix norm for TSS matrix at every instance. Each TSS matrix corresponds to all TSS between words belonging to the same category (qualified and nonqualified) at a given time. From the time before the draw (D − 3) until the draw, the qualified and nonqualified teams show a correlated behavior (Figure 4(d), *ρ* = 0.50919, *p* < 10^−3^). Starting from the instant just after the draw (D + 1) until the instant where the two series stop decreasing, these series show a stronger correlation (*ρ* = 0.71126, *p* < 10^−9^). In the remaining time, the series are also correlated (*ρ* = 0.42327, *p* < 10^−3^). This shows that the World Cup draw modified the expected network (qualified teams) but also the nonqualified teams. This behavior evidenced the distributed effect on the network. During March 2014, the series for the qualified and nonqualified teams were not correlated showing that both subnetworks stabilize to a basal movement, not orchestrated by the particular event.

## 4. Conclusions

Many unsupervised algorithms explore semantic organization based on frequency of cooccurrence of words from large corpora. Semantic spaces are computationally expensive to calculate, prohibiting the estimation of semantic networks in short periods of times. Instead, we study activity in the social network to drive a measure of the underlying semantic space and compare it with previous metrics.

With TSS, we have presented a measure of similarity with many features that make it a valuable tool to study semantic structures. We demonstrated that TSS is commensurable with methods, for example, LSA, Wordnet, that assume a stationary or slowly varying field of cooccurrences between the elements of the lexicon. We validated TSS in several tests, such as categorization or synonym test, which yielded similar results, and therefore it can be used to substitute them in cases that do not involve a highly specialized semantic space (e.g., a professional field). Conversely, TSS allows defining semantics using more colloquial language expressions, and therefore it can be thought of as a vernacular database, sensitive to slang and the emergence of neologisms [33].

However, the most remarkable feature of TSS is its ability to detect rapid changes in the semantic network without relying on specific topics and to connect those changes with real-time events across the world. We quantified the effect of these events on the semantic content of terms directly related to them, as well as the ripple effect across the semantic web. This is particularly important, as the definition of TSS does not involve any explicit assumption about the distributed nature of semantics, in contraposition with semantic indexing and graph-based methods such as [34, 35].

The idea that our beliefs are dynamic is both common-sensical and deeply rooted in psychology [36] and philosophy of language [37], as well as machine learning and artificial intelligence theories of relational learning [38]. With the advent of social media, there is growing interest in the dynamics of information diffusion [39, 40]. Using TSS, we showed that seemingly permanent lexical elements, such as terms designing countries, are affected semantically by the news/event dynamics. While our results should be considered limited and preliminary, they are consistent with the notion that language emerges from a complex web of* dynamically* interacting elements. In this sense, we hope our work will contribute to the development of a new formal paradigm to understand language, reflecting its nature as a social construction [41].

## Supplementary Material

Supplemental material compares TSS measure to Joint Frequency. JF showed a strong correlation with TSS, but TSS showed higher performance in the evaluation datasets.
