# Supplementary material for: Fast Distributed Dynamics of Semantic Networks via Social Media
Source: Comput Intell Neurosci. 2015 May 17;2015:712835. doi: 10.1155/2015/712835 (PMC4449913; doi:10.1155/2015/712835)
Supplement: Supplementary file 1 — Supplemental material compares TSS measure to Joint Frequency. JF showed a strong correlation with TSS, but TSS showed higher performance in the evaluation datasets. [file 712835.f1.pdf]

Fast Distributed Dynamics of Semantic Networks via Social Media (Supplemental Material)

Facundo Carrillo[1], Guillermo A. Cecchi[3], Mariano Sigman[2], and Diego Fernández Slezak[1]

[1] Laboratorio de Inteligencia Artificial Aplicada, Depto. de Computación, Ciudad Universitaria, 1428 Buenos Aires, Argentina
[2] Universidad Torcuato Di Tella, Ciudad Autónoma de Buenos Aires, Argentina
[3] Computational Biology Center, T.J. Watson Research Center, IBM, P.O. Box 218, Yorktown Heights, N.Y. 10598, USA.

1 TSS vs Joint Frequency

There are a lots of works where Twitter is used as a corpus to understand different phenomenons. However, these works have been used the frequency or the amount of tweets as a principal source. In our work we upgraded this approach using not just the frequency of a term else the correlation between them. As we showed in Methods the correlation among two terms is computed by using the frequency of the tweets with the two terms and the frequency of each single term (see Methods for the equation). This approach has advantages when its compared with the use of the simple frequency between terms (i.e. correlation formula without the normalization of the frequencies of each term). Using some examples of the Result section, here, we showed how the TSS works compared to the *joint frequency* (JF).

We compared TSS vs two typically semantic networks, Latent Semantic Analysis and Wordnet (see Stationary semantic organization via TSS in Results section). For this, we set up a list of 102000 pairs of nouns and computed their similarity in TSS, LSA and Wordnet. TSS showed a good correlation with both similarities ($\rho = 0.2199$ and $\rho = 0.0784$ respectively). Following this schema we used JF to understand how works compared with TSS, LSA and Wordnet. JF showed a strong correlation with TSS ($\rho = 0.23081$, pval=0), the correlation with LSA and TSS decreased significantly ($\rho = 0.024522$ and $\rho = 0.0096175$ respectively). This showed the the normalization in the TSS formula improve the similarity with a well-tested metrics.

Other tests we performed in the result section was to verified that TSS recognize semantic clusters . For this we setup three categories of intuitive easy words (see Common semantic categories in Methods section). With the selected words we performed the TSS between each words. This similarity matrix allows us to classified using 10 folds cross-validation. Using TSS and RandomForest algorithm [1] we obtained the best performance, above 88%. Using the same configuration, with JF similarity matrix we obtained a performance of 42%.

The last test we performed for stationary semantic organization using TSS was the analysis of the world organization. For this, we selected the English words of countries of Asia, America, Africa and Europe and measure their TSS Matrix. We showed that a two dimensional projection TSS Matrix allows us to identify to a large extent continental and geographical organization of the countries. To quantified this effect we ran a classifier to see whether TSS can be used to infer the continent to which a country belongs (10 folds cross-validation, K* algorithm) and we obtained a performance above 90%. With the same analysis using JF similarity Matrix we obtained a performance of 64%.

References

1. Leo Breiman. Random forests. *Machine learning*, 45(1):5–32, 2001.
